# Hierarchical Mn_2_O_3_ Microspheres In-Situ Coated with Carbon for Supercapacitors with Highly Enhanced Performances

**DOI:** 10.3390/nano7120409

**Published:** 2017-11-23

**Authors:** Feilong Gong, Shuang Lu, Lifang Peng, Jing Zhou, Jinming Kong, Dianzeng Jia, Feng Li

**Affiliations:** 1 Institute of Applied Chemistry, Xinjiang University, Urumqi 830046, China; feilonggong@zzuli.edu.cn (F.G.); ls102511@163.com (S.L.); 18736048695@163.com (L.P.); jzhou_chem@zzuli.edu.cn (J.Z.); jdz@xju.edu.cn (D.J.); 2 State Laboratory of Surface and Interface Science and Technology, Zhengzhou University of Light Industry, Zhengzhou 450002, China; 3 School of Environmental and Biological Engineering, Nanjing University of Science and Technology, Nanjing 210094, China; j.kong@njust.edu.cn; 4 American Advanced Nanotechnology, Houston, TX 77459, USA

**Keywords:** Mn_2_O_3_ microspheres, in-situ carbonization, amorphous carbon, hierarchical materials, pseudocapacitors

## Abstract

Porous Mn_2_O_3_ microspheres have been synthesized and in-situ coated with amorphous carbon to form hierarchical C@Mn_2_O_3_ microspheres by first producing MnCO_3_ microspheres in solvothermal reactions, and then annealing at 500 °C. The self-assembly growth of MnCO_3_ microspheres can generate hollow structures inside each of the particles, which can act as micro-reservoirs to store biomass-glycerol for generating amorphous carbon onto the surfaces of Mn_2_O_3_ nanorods consisting of microspheres. The C@Mn_2_O_3_ microspheres, prepared at 500 °C, exhibit highly enhanced pseudocapacitive performances when compared to the particles after annealed at 400 °C and 600 °C. Specifically, the C@Mn_2_O_3_ microspheres prepared at 500 °C show high specific capacitances of 383.87 F g^−1^ at current density of 0.5 A g^−1^, and excellent cycling stability of 90.47% of its initial value after cycling for 5000 times. The asymmetric supercapacitors assembled with C@Mn_2_O_3_ microspheres after annealed at 500 °C and activated carbon (AC) show an energy density of up to 77.8 Wh kg^−1^ at power density of 500.00 W kg^−1^, and a maximum power density of 20.14 kW kg^−1^ at energy density of 46.8 Wh kg^−1^. We can attribute the enhanced electrochemical performances of the materials to their three-dimensional (3D) hierarchical structure in-situ coated with carbon.

## 1. Introduction

Supercapacitors, as old brothers of batteries, have attracted considerable attentions in the last decade, because of the dramatic advances concerned with materials science and the theories of storing charges [1,2,3,4]. The capacitive devices developed recently, which possess excellent electrochemical performances with a high power density, long cycle stability, high safety, and improved energy density, have been applied in consumer electronics and electric vehicles [5]. In order to design and fabricate advanced energy storage devices, Mn-based materials, such as MnO_2_, Mn_2_O_3_, and Mn_3_O_4_, are promising candidates in assembling electrodes for their high capacities in theory, low cost, rich in reserves, and low environmental foot-print [6,7,8,9]. Their applications in storing energy, however, suffer from their intrinsically low conductivity. It has been therefore investigated extensively to improve the electrochemical performances of the materials through constructing hierarchical composites with conductive materials, including grapheme [10], carbon nanotubes [11], carbon aexogel [12], mesoporous carbon [13], biocarbon [14], active carbon [15], carbon fibers [16], and conductive polymers [17] in recent years. When compared with the extensive researches concerned with energy storage devices made with MnO_2_ [18,19,20], however, it is relatively rare in literature concerned with the supercapacitors made with Mn_2_O_3_-based materials so far [21,22,23,24].

**Scheme 1:**
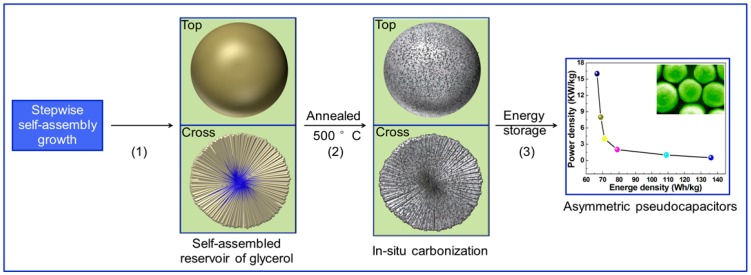
The in-situ carbonization of glycerol on the surfaces of porous Mn_2_O_3_ microspheres for asymmetric pseudocapacitors. (1) Stepwise self-assembly growth of glycerol reservoir in MnCO_3_ microspheres; (2) in-situ carbonization to produce amorphous carbon layers on the surfaces of Mn_2_O_3_ microspheres; and (3) asymmetric supercapacitors.

As to materials for storing energy, both their morphology and size, are critical in affecting their practical applications. It is highly desired to produce hierarchical electrode materials with controlled shape, porous structures, and size for consistently assembling advanced electrodes with high uniformity and density. The researches in our groups have mainly focused on construct nanoarchitectures with nanosized building blocks for tailoring their functionalities [25,26,27,28,29,30]. Monodispersed ZnO supercrystals with anisotropic blue emission, for instance, can be produced with an approach based on stepwise self-assembly growth [27]. The voids in-situ generated inside the supercrystals could be applied to store biomass as reservoirs for coating conductive carbon layer onto their surfaces effectively, and therefore improving their electrochemical performances. Herein, we would like to report a novel synthesis and highly enhanced electrochemical properties of hierarchical C@Mn_2_O_3_ microspheres based on this idea (Scheme 1). Uniform precursor MnCO_3_ microspheres with tunable diameter can be first produced on a large scale with a solvothermal approach in a mixed solvent consisting of glycerol and water. It was found that biomass-glycerol can be encapsulated in the voids inside MnCO_3_ microspheres to form micro-reservoirs during their growth. Hierarchical Mn_2_O_3_ microspheres in-situ coated with amorphous carbon can be finally produced by annealing the precursors carrying biomass. It is found that glycerol has played a critical role in the synthesis of the hierarchical C@Mn_2_O_3_ microspheres, not only as solvent to conduct the reactions producing uniform precursor MnCO_3_ microspheres, but also as biomass to generate carbon layers onto the surfaces of final products. The Mn_2_O_3_ microspheres in-situ coated with carbon show highly enhanced pseudocapacitive properties, which can be attributed to the hierarchical structures of the microspheres. Specifically, a new pathway for decorating Mn-based electrode materials with carbon layers is opened by simply employing biomass glycerol as solvent in the synthesis and its in-situ carbonization at a higher temperature, subsequently. The materials can be applied to assemble advanced electrodes for storing energy.

## 2. Results

**Figure 1:**
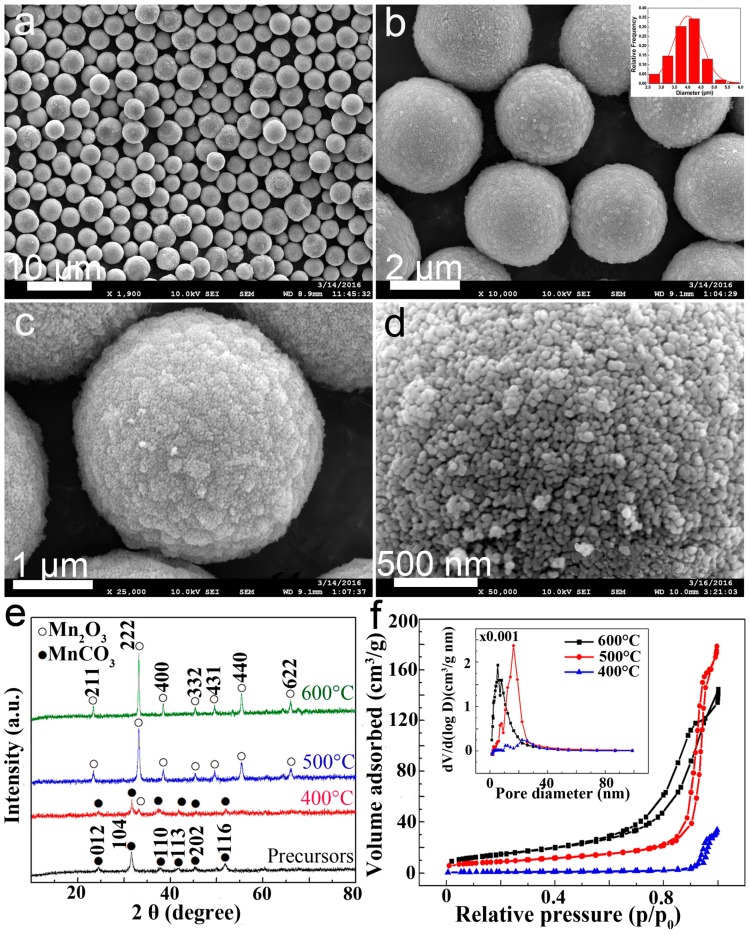
(**a**–**d**) Field emission scanning electron microscopy (FESEM) images of C@Mn_2_O_3_-500. (**e**) XRD profiles of the precursors before and after annealed at 400, 500 and 600 °C, respectively, and (**f**) N_2_ adsorption-desorption (BET) curves of C@Mn_2_O_3_-400, C@Mn_2_O_3_-500 and C@Mn_2_O_3_-600. The inset in (**b**) presents the diameter distribution of the microspheres. The insets in (**f**) show the pore size distributions of C@Mn_2_O_3_-400, C@Mn_2_O_3_-500 and C@Mn_2_O_3_-600.

The as-prepared C@Mn_2_O_3_-500 materials were first characterized with field emission scanning electron microscopy (FESEM), as shown in Figure 1a–d. Microspheres with high uniformity and regularity can be produced on a large scale. The inset in Figure 1b shows narrow size distribution of the materials of 3.90 ± 0.55 µm in diameter. A lot of pores of ca. 20 nanometers can be observed clearly on the surfaces of the microspheres (Figure 1c,d). Figure 1e presents the XRD profiles of C@Mn_2_O_3_-400, C@Mn_2_O_3_-500, and C@Mn_2_O_3_-600. The XRD pattern of the precursors, consists of peaks that can be attributed to the (012), (104), (110), (113), (202), and (116) facets of MnCO_3_ (JCPDS No. 44-1472). After being treated at 400 °C for 8 h, most of the diffraction peaks in the XRD profile can be also attributed to MnCO_3_, while the intensity of the MnCO_3_ diffractions decrease dramatically and a new broaden peak corresponding to (222) plane of Mn_2_O_3_ appears at 33.08°. The results indicate that MnCO_3_ precursors start to decompose and convert to Mn_2_O_3_ at 400 °C. In contrast, the XRD profile of materials only consists of diffraction peaks that are attributed to Mn_2_O_3_, after being annealed at 500 °C. After being treated at 600 °C, the intensities of diffraction peaks that are attributed to Mn_2_O_3_ further increase. The diffraction peaks corresponding to (211), (222), (400), (322), (431), (440), and (662) facets of bixbyite Mn_2_O_3_ (JCPDS No. 41-1442) can be observed clearly. Porous Mn_2_O_3_ microspheres with highly crystalline nature can be produced by annealing MnCO_3_ precursors at higher than 400 °C. The porous structures existing throughout the C@Mn_2_O_3_ microspheres can be ascribed to the decomposition of MnCO_3_ precursors and the release of CO_2_ out of the materials. Mn_2_O_3_ microspheres can be produced successfully on a large scale by annealing as-prepared precursors at 500 °C. There is not any diffraction peak from other crystalline impurity in the XRD profiles (XRD deviation of 5%). The porous structures of the materials can be further characterized with BET measurements, as shown in Figure 1f. The nitrogen adsorption and desorption isotherms of the materials exhibit a typical H3 hysteresis loop, indicating the presence of aggregated particles with slit shape pores. The calculated BET surface area of C@Mn_2_O_3_-500 of 30.68 m^2^ g^−1^, based on the adsorption and desorption isotherms, is lower than 50.81 m^2^ g^−1^ of C@Mn_2_O_3_-400, but much higher than 2.50 m^2^ g^−1^ of C@Mn_2_O_3_-600. The pore diameter of C@Mn_2_O_3_-400 concentrates on ~8.2 nm, it increases to ~18.5 nm in C@Mn_2_O_3_-500. However, there are abundant irregularpores of 2–100 nm in diameter existing in the systems. The average pore diameters of the materials are 12.1, 20.2, and 31.6 nm, after being annealed at 400, 500, and 600 °C, respectively. The much lower BET value and non-uniform pores in C@Mn_2_O_3_-600 should be caused by thecollapse and the aggregation of nanoparticles composed of the microspheres. It is worth-noting that the yield (107.5 ± 2.5%) of C@Mn_2_O_3_-500 is higher than 100%, based on the total conversion of reactant Mn(Ac)_2_·4H_2_O to Mn_2_O_3_. The unexpected high yield implies that amorphous materials could be also produced in the system.

**Figure 2:**
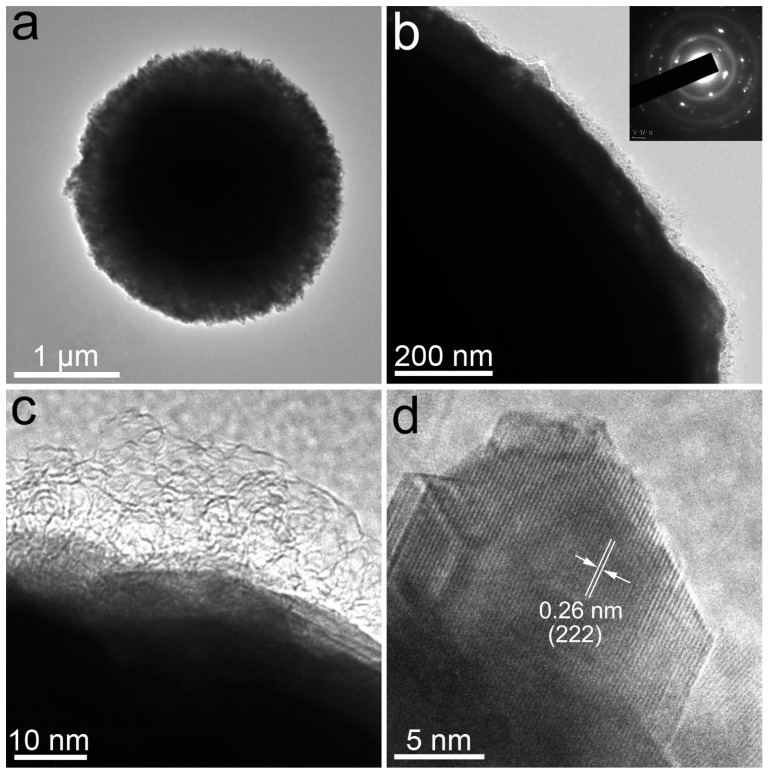
(**a**–**c**) TEM and (**d**) HRTEM images of C@Mn_2_O_3_-500 microsphere after annealed at 500 °C for 8 h. The inset in (**b**): selective area electron diffraction pattern.

More details that are concerned with the microstructures of C@Mn_2_O_3_-500 can be further revealed by TEM observations. The TEM image of a microsphere (Figure 2a) at low magnification shows its porous interior. The higher magnification TEM image (Figure 2b) of the microsphere collected at its edge highlights its surface that is covered with a thin layer of ca. 15 nm in thickness. The selective area electron diffraction pattern (inset in Figure 2b) of the microsphere is composed of random dots and rings attributed to polycrystalline Mn_2_O_3_ and amorphous carbon, respectively. After further enlarging the area at the edge of the microsphere (Figure 2c), we can directly observe an amorphous carbon layer of 15 nm in thickness on its surface. The carbon coatings on the surfaces of the microspheres can be attributed to their high yield of more than 100%. The high resolution transmission electron microscopy (HRTEM) image (Figure 2d) taking at the edge of a microsphere consists of d-spacing of 0.26 nm corresponding to (222) plane of Mn_2_O_3_. Porous and uniform C@Mn_2_O_3_-500 microspheres that are composed of nanoparticles of ca. 20 nm in diameter can be produced successfully by directly annealing MnCO_3_ precursors at 500 °C. It is interesting to find that amorphous carbon layers can be produced on the surfaces of the microspheres, while there is not any carbon resource added into the precursor before annealing. We can therefore suggest two ways that could be related to the formation of the carbon layers: pre- or during the decomposition of the precursors.

**Figure 3:**
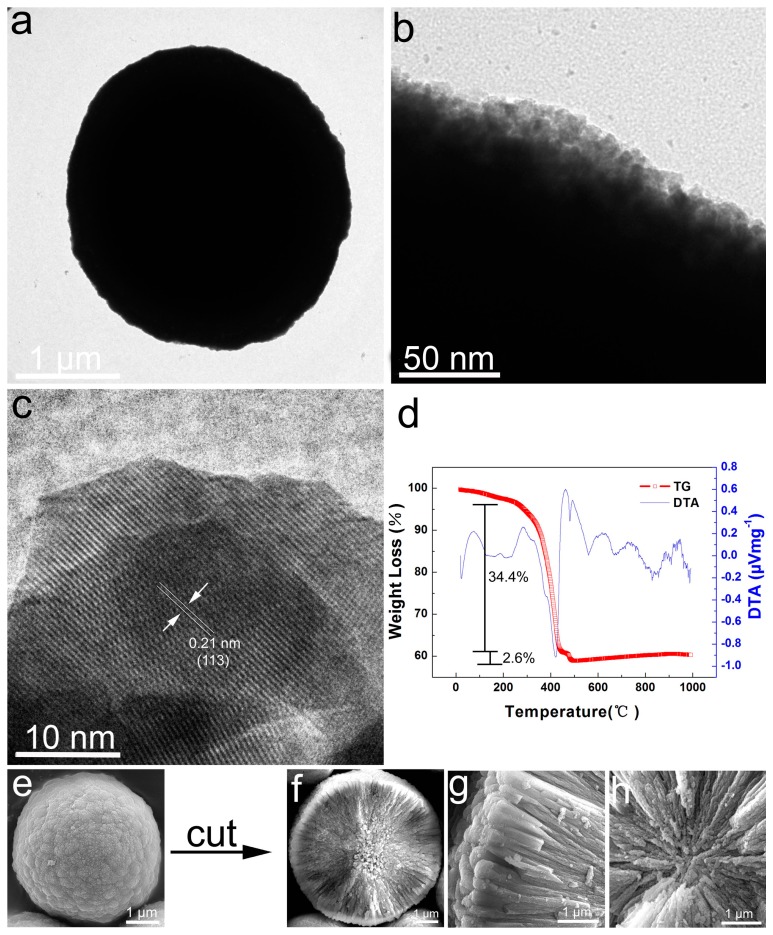
(**a**,**b**) The TEM, (**c**) HRTEM images, and (**d**) TG-DTA curves of MnCO_3_ microspheres. (**e**) SEM image of one precursor microsphere, (**f**) top view of a half-microsphere, (**g**) edge, and (**h**) central areas of a half-microsphere.

The TEM image of aMnCO_3_ microsphere (Figure 3a,b), however, shows much darker contrast and clean surfaces when compared with C@Mn_2_O_3_-500 microspheres (Figure 2a). There is no amorphous material existing on the surfaces of the particles. The results reveal that the carbon coatings on the surfaces of the C@Mn_2_O_3_ microspheres should be produced during their annealing process instead of in the reactions. The HRTEM image (Figure 3c) of the precursor is composed of d-spacing of 0.21 nm, which corresponds to (113) plane of MnCO_3_. In order to investigate what has resulted in the formation of carbon layers on the surfaces of the microspheres, thermogravimetry and differential thermal analysis (TG-DTA) (Figure 3d) of the precursors were performed. The precursors lost their weight of 4.2% continually before 200 °C for the surface water absorbed on the materials. The two shoulder exothermal peaks that appeared at 321.3 and 382.1 °C in the differential thermal analysis (DTA) curve can be attributed to biomass glycerol decomposing and polymerizing exothermically [31]. The MnCO_3_ precursor then decomposes and converts into Mn_2_O_3_ completely at 427 °C, with a weight loss of 34.4% for the complete decomposition of carbonate group and release of CO_2_ from materials (calculated weight loss: 31.3%). The actually 3.1% higher weight loss of the precursor in comparison with the calculated value in theory could be resulted from the decomposition and polymerization of glycerol at 321.3 and 382.1 °C, respectively. It is very interesting to observe another weight loss of 2.6% at 479 °C, which can be attributed to the carbonization and the formation of carbon layers on the surface of the Mn_2_O_3_ microspheres [31]. The TG-DTA results further verify that porous Mn_2_O_3_ materials can be generated by releasing CO_2_ out of the precursors. The higher weight loss of the system in comparison with the one that is calculated in theory can be ascribed to the decomposition of biomass. Since there is not any carbon resource other than glycerol getting involved in the reactions, the formation of carbon layers on the surfaces of microspheres can be attributed to its carbonization during the annealing process. The TG-DTA results are consistent with those from the XRD profiles and the high yield of the products. In addition, the SEM image (Figure 3e) of a whole precursor microsphere shows a dense surface when compared to porous C@Mn_2_O_3_-500 microspheres. However, the top-views (Figure 3f–h) of half-microspheres clearly reveal the porous interiors of the microspheres. The dynamic structure evolutions of the microspheres corresponding to reaction time and temperature are presented in Figures S1 and S2, which indicate that the formation of uniform MnCO_3_ microspheres is very similar to the stepwise self-assembly growth of ZnO twin-spheres [27]. The one dimensional (1D) nano-wedges can self-assemble to form hierarchical microspheres with voids inside (Scheme 1). The biomass glycerol, which can be trapped in the voids inside the precursor microspheres during their growths to form carbon reservoirs, can generate carbon layers on the surfaces of the microspheres during annealing.

**Figure 4:**
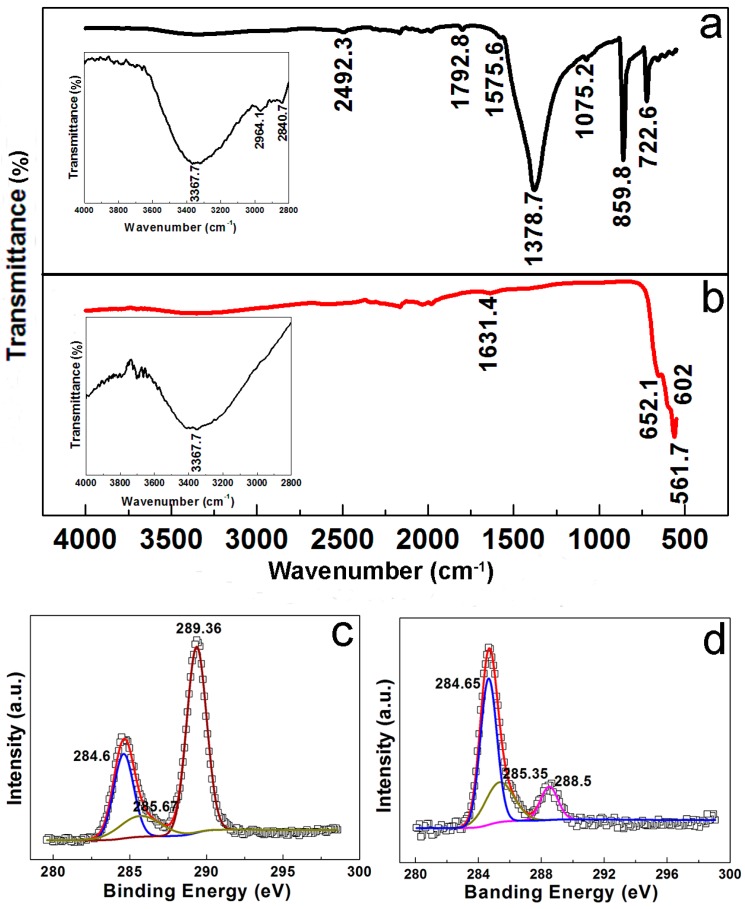
FTIR spectra of (**a**) the precursor and (**b**) C@Mn_2_O_3_-500. X-ray photoelectron spectroscopy (XPS) C1s spectra of (**c**) the precursor and (**d**) C@Mn_2_O_3_-500. The insets in (**a**,**b**) highlight the FTIR spectra of precursors and final products from 2800–4000 cm^−1^ with enlarged vertical axis, respectively.

In order to further understand the formation of carbon layers on the surfaces of products, it is necessary to verify the presence of glycerol trapped in as-prepared precursor microspheres. Figure 4a presents a typical Fourier transformed infrared (FTIR) spectrum of MnCO_3_ precursors. The bands at 3367.7 and 1575.6 cm^−1^ can be assigned to the stretching vibration of O–H group attributed to surface absorbed molecular, such as water and glycerol. The presence of CO_3_^2−^ in MnCO_3_ is evidenced by its fingerprint peaks at 1378.7, 859.8, and 722.6 cm^−1^, in according to normal modes of vibration of planar CO_3_^2−^ ions [1]. The peak located at 2492.3 cm^−1^ is also commonly associated to the vibration mode of carbonate anion. The weak peak at 1792.8 cm^−1^ is attributed to an overtone or combination band of carbonate groups and divalent metal ions. However, the weak peaks at 2964.1 and 2840.7 cm^−1^ (inset in Figure 4a) can be undoubtedly assigned to the stretching vibration of C–H bond that is attributed to glycerol. A weak peak appearing at 1075.2 cm^−1^ can further confirm the stretching vibration of C–O bond of glycerol. The FTIR spectrum of the precursor clearly verifies that glycerol has been trapped inside the precursor microspheres. In contrast, the characteristic peaks that are attributed to MnCO_3_ and glycerol all disappear in the FTIR spectrum (Figure 4b) of final products after being annealed at 500 °C. The three characteristic peaks located at 561.7, 602.0, and 652.1 cm^−1^ can be assigned to Mn–O stretching vibrations of Mn_2_O_3_. However, a weak peak located at 1634 cm^−1^ attributed to C=O group still appears in the spectrum, which indicate the presence of residue carbon resulted from the carbonization of glycerol.

The precursors and C@Mn_2_O_3_-500 were further characterized with XPS carefully to verify the presences of carbon (Figure 4c,d and Figure S3) in the products. In the C-1s spectrum (Figure 4c) of the precursor, the peak located at 289.36 eV can be assigned to the carbon element that is associated with oxygen in the carbonate ions. It disappears in the C-1s spectrum of final product (Figure 4d) for the decomposition of MnCO_3_ and the formation of Mn_2_O_3_. The peaks at 285.67 eV and 285.35 eV in Figure 4c,d can be generally attributed to surface-adsorbed hydrocarbons and their oxidative forms (e.g., C–OH). In the C-1s spectrum of final product, however, a new peak appearing at 288.5 eV can be assigned to the group of C-O-C, which directly reveals the presence of amorphous carbon on the surface of the materials. The XPS spectra further confirm the presence of residue carbon in the final products. It was reported that biomass glycerol starts to decompose and form polyglycerol from 200 to 380 °C, and then converts into carbon materials at ca. 500 °C [31]. Our experimental results verify that porous and uniform Mn_2_O_3_ microspheres that are coated with amorphous carbon can be produced on a large scale by annealing MnCO_3_ precursors with biomass glycerol in-situ encapsulated inside the voids of the particles.

**Figure 5:**
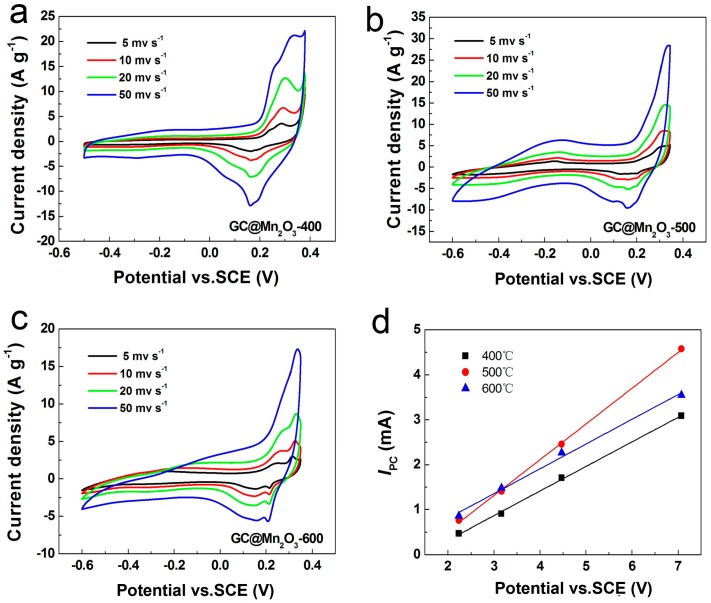
CV curves of (**a**) C@Mn_2_O_3_-400, (**b**) C@Mn_2_O_3_-500, and (**c**) C@Mn_2_O_3_-600 measured in three-electrode system in KOH electrolytes at different scan rates. (**d**) The relationship between the cathodic peak current (*I*_pc_) and the square root of the scan rate (ν^1/2^) of the GC@Mn_2_O_3_-400. GC@Mn_2_O_3_-500 and GC@Mn_2_O_3_-600.

**Figure 6:**
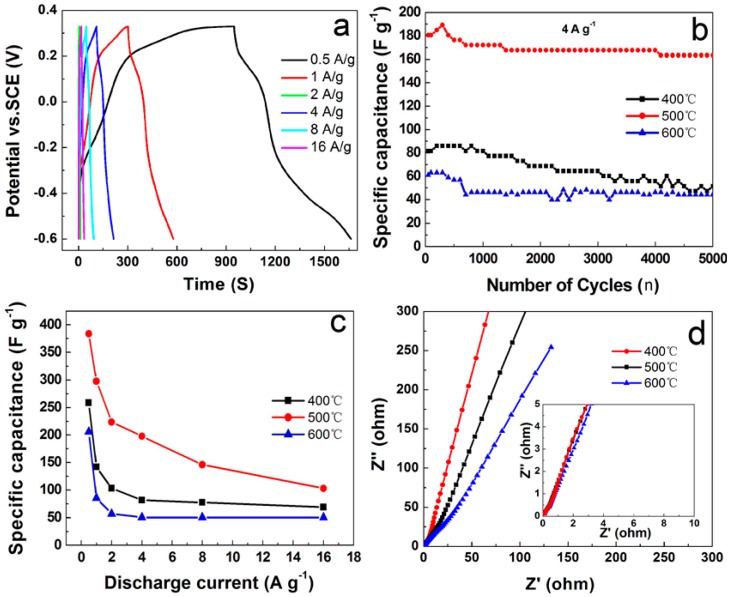
Electrochemical performances of C@Mn_2_O_3_-400, (**b**) C@Mn_2_O_3_-500, and (**c**) C@Mn_2_O_3_-600 measured in three-electrode system in KOH electrolytes. (**a**) charge–discharge curves at a current density of 0.5 A g^−1^, (**b**) specific capacitance versus cycle number of the materials at a current density of 4 A g^−1^, (**c**) average specific capacitances at different current densities, and (**d**) typical Nyquist impedance (EIS) spectra.

The porous and uniform C@Mn_2_O_3_ microspheres of 3.90 ± 0.55 µm in diameter prepared by annealing the precursors at 500 °C were applied to assemble working electrodes with Ni foams to investigate their energy storage performances in a three-electrode configuration first. Figure 5 shows the Cyclic Voltammogram(CV) curves of the electrodes made with C@Mn_2_O_3_-400, C@Mn_2_O_3_-500, and C@Mn_2_O_3_-600 in KOH electrolyte at scan rates ranging from 5 to 100 mV s^−1^ in their own potential window of −0.5–+0.35, −0.6–+0.35, and −0.6–+0.35 V (vs. SCE), respectively. The distinct redox peaks at 0.27 and 0.13, −0.156, and 0.13, and 0.27, and 0.21 V in the CV curves of as-prepared three materials indicate the Faradic pseudocapacitive nature of the electrodes. As the scan rate increases, the current subsequently increases while the shape of the CV curves keeps almost unchanged. This strongly demonstrates the fast electrons and ions diffusion rate in the electrodes made with C@Mn_2_O_3_ microspheres. Moreover, an almost linear/quasi-linear relationship for the GC@Mn_2_O_3_-400, GC@Mn_2_O_3_-500 and GC@Mn_2_O_3_-600 electrodes is observed between cathodic peak current and applied scan rate in Figure 5d, indicating that the reactions in the system are controlled by the diffusion process (bulk chargestorage) and surface redox reactions taking place in the charge storage process [32,33]. The electrochemical properties of the electrodes are further presented in Figure 6 and Figures S5 and S6. According to the discharge time in charge/discharge curves (Figure 6a, Figures S6a and S7a), the specific capacitances (Figure 6c) of the electrodes made with materials annealed at 500 °C are much higher than those of treated at 400 and 600 °C, respectively. Specifically, GC@Mn_2_O_3_-500 always shows the highest specific capacitances of 383.87, 297.85, 223.66, 197.85, 146.24, and 103.23 F g^−1^ at current density of 0.5, 1, 2, 4, 8, and 16 A g^−1^, respectively, as compared to GC@Mn_2_O_3_-400 and GC@Mn_2_O_3_-600. The specific capacitances of all of the materials decrease with the increase of current density, which could be resulted from the increase of internal diffusion resistance within the pseudocapacitive material, and therefore the decreases of utilizing the active materials efficiently. Figure 6b shows the cycling stabilities of the electrodes by conducting charge/discharge tests at a current density of 4 A g^−1^. The C@Mn_2_O_3_-500 electrode can maintain 90.47% of its initial value even after cycling for 5000 times, which shows the excellent cycling stability of the materials. The EIS spectra (Figure 6d) of the three materials further demonstrate that they possess the minimum radius of semicircles in the high frequency region and their slopesare all higher than 45° in the low frequency regions. The results indicate that Warburg resistance is not the determinable factor and the electrodes can store charges efficiently. The electrodes made with C@Mn_2_O_3_-500 exhibit enhanced electrical conductivity. The results indicate that the annealing temperature can dramatically affect the electrochemical performances of the materials. This can be attributed to the composition, pore structures and surface carbon coating of the materials, which are all closely related to the reaction temperature. Firstly, the biomass-glycerol trapped in the voids of the microspheres can be carbonized at 479 °C to form carbon thin films on the surfaces of porous Mn_2_O_3_ microsphere. Secondly, after being annealed at 500 °C, the materials also have a much higher BET surface area (Figure 1f) in comparison with those after being annealed at 600 °C. The third, MnCO_3_ can convert to Mn_2_O_3_ completely after annealed at 500 °C, as compared to C@Mn_2_O_3_-400 consisting of MnCO_3_ mainly for its decomposition at 427 °C. The C@Mn_2_O_3_-500 electrodes therefore exhibit highly enhanced capacitive performances when compared to C@Mn_2_O_3_-400 and C@Mn_2_O_3_-600. The highly enhanced electrochemical performances of C@Mn_2_O_3_-500 electrodes can be ascribed to the optimized composition and hierarchical structure with surface carbon coating, which can improve both the electron transportation and ion diffusion.

**Figure 7:**
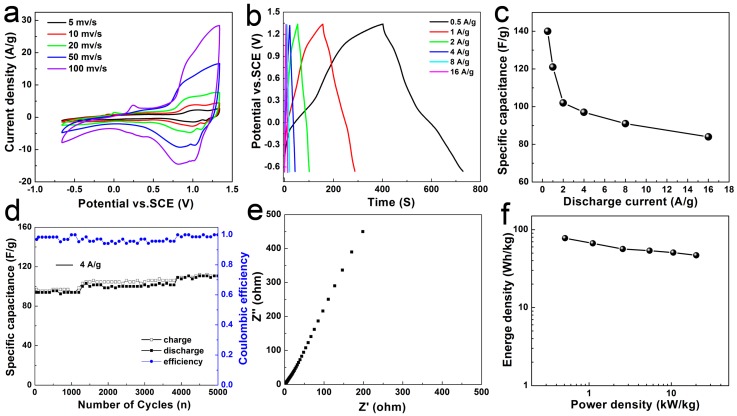
Electrochemical performances of C@Mn_2_O_3_-500//active carbon (AC) asymmetric supercapacitors. (**a**) Cyclic Voltammogram (CV) curves at different scan rates; (**b**) charge/discharge curves at different current densities; (**c**) the specific capacitances at different current densities; (**d**) cycle stability and Coulombic efficiency in KOH electrolyte of 6 M; (**e**) Nyquist impedance spectrum; and (**f**) Ragone plots.

In order to further evaluate the energy storage performances of the materials, asymmetric supercapacitors (Figure 7) were assembled by using C@Mn_2_O_3_-500 as positive electrode materials and AC as negative electrode materials. Figure 7a shows the CV curves of C@Mn_2_O_3_-500//AC asymmetric supercapacitors measured in KOH (6 M) electrolyte solution at scan rates of 5, 10, 20, 50, and 100 mV s^−1^, respectively. Two types of redox peaks can be observed in the CV curves of C@Mn_2_O_3_-500//AC. Moreover, the potential window of C@Mn_2_O_3_-500//AC asymmetric supercapacitors increases to 2 V, which is almost twice that of conventional capacitors made with active carbon (AC) in aqueous electrolytes (Figure S4). Figure 7b shows galvanostatic charge/discharge curves of the asymmetric supercapacitor at various current densities. In according to the discharge curves of C@Mn_2_O_3_-500//AC cells, the calculated specific capacitances (Figure 7c) based on the total mass of active materials are 140, 121, 102, 97, 91, and 84 F g^−^^1^ at current density of 0.5, 1, 2, 4, 8, and 16 Ag^−1^, respectively. The cycling stability of C@Mn_2_O_3_-500//AC asymmetric pseudocapacitors was further investigated by galvanostatic charge/discharge cycling between 0 and 2 V at a current density of 4 Ag^−1^. A total number of 5000 cycles was continuously performed and the variations of specific capacitance of the cells in accompanying with cycling the tests were shown in Figure 7d. The specific capacitances of 97 F g^−1^ keep stable at first 300 cycles, then increase ca. 1.6%, and finally return and stay at 106.1 F g^−1^ until 5000 cycles. When compared to its initial value, the device can retain 109.4% of its specific capacitance value after cycling for 5000 times. The EIS spectrum (Figure 7e) of the device further indicates that it possesses a minimum radius of semicircles in the high frequency region and its slope is higher than 45° in the low frequency region. This confirms that the Warburg resistance is not the determinable factor and that the electrode can store charges more efficiently. The electrodes made with C@Mn_2_O_3_-500 also show enhanced electrical conductivity.

To further evaluate the electrochemical performances of the asymmetric cells, Ragone plot (Figure 7f) relative to the corresponding energy (*E*) and power (*P*) densities of the devices was calculated by following Equations (4) and (5). The asymmetric capacitors exhibit the highest energy density of 77.8 Wh kg^−1^ at power density of 500.00 W kg^−1^ and a maximum power density of 20.14 kW kg^−1^ at an energy density of 46.8 Wh kg^−1^. The electrochemical performances of hierarchical microspheres are superior to the Mn_2_O_3_ materials reported so far [34], and the energy density of the device assembled with C@Mn_2_O_5_-500 is also much higher in comparison with that of devices made with Mn-based electrode materials such as MnO_2_/AC [13], MnO_2_/GO [35,36], Mn_3_O_4_/Carbon-aerogel [12], MnOOH/GO [25], MnO_2_/Carbon-nanofiber [16], MnO_2_/AC [37], MnO_2_/Onion-like-carbon [15], MnO_2_/CNT [20,38], and Mn_3_O_4_/RGO [28] in aqueous electrolyte solutions. The carbon layers in-situ coated onto the surfaces of hierarchical Mn_2_O_3_ microspheres may have played a key role in highly improving their electrochemical performances. The hierarchical C@Mn_2_O_3_ microspheres are promising candidates that are served as electrode materials for energy storage.

## 3. Discussion

Based on a stepwise self-assembly growth approach, monodispersed MnCO_3_ precursor microspheres with well-controlled size and morphology can be first produced in glycerol, and then converted into hierarchical porous Mn_2_O_3_ microspheres that are coated with amorphous carbon on a large scale by annealing at 400, 500, and 600 °C for 8 h. We can decorate Mn-based electrode materials with carbon layers through simply employing glycerol as solvent in the synthesis and then in-situ carbonize the biomass at higher temperature subsequently. Based on TG-DTA, TEM, FTIR, and XPS results, we found that the biomass glycerol, which is employed as solvent to perform the reactions, can be encapsulated in the voids inside the precursor microspheres to form micro-reservoirs for in-situ generating carbon layers on the surfaces of final products to form hierarchical C@Mn_2_O_3_ microspheres. Finally, when compared with C@Mn_2_O_3_-400 and C@Mn_2_O_3_-600, C@Mn_2_O_3_-500 shows highly enhanced electrochemical performances both in three-electrode and asymmetric systems. The specific capacitances of the electrodes made with materials after annealed at 500 °C are much higher than those treated at 400 and 600 °C, respectively. Specifically, GC@Mn_2_O_3_-500 always shows the highest rate and capacitances of 383.87, 297.85, 223.66, 197.85, 146.24, and 103.23 F g^−1^ at current density of 0.5, 1, 2, 4, 8, and 16 A g^−1^, respectively, as compared to GC@Mn_2_O_3_-400 and GC@Mn_2_O_3_-600. The asymmetric supercapacitors assembled with C@Mn_2_O_3_-500 microspheres and active carbon (AC) exhibit high energy density of 77.8 Wh kg^−1^ at a power density of 500.00 W kg^−1^, and a maximum power density of 20.14 kW kg^−1^ at energy density of 46.8 Wh kg^−1^. The highly enhanced electrochemical performances of the materials can be attributed to their surface carbon coatings.

## 4. Materials and Methods

All of the reagents were analytically pure and were purchased from Shanghai Chemical Industrial Co. Ltd. (Shanghai, China), and were used as received.

### 4.1. Preparation of C@Mn_2_O_3_Microspheres

Typically, Mn(Ac)_2_·4H_2_O (0.6127 g, 2.5 mmol) and carbamide (0.3000 g, 5 mmol) were dissolved in a mixed solvent of water (10.0 mL) and glycerol (30.0 mL). The solution was then transferred to a Teflon-lined stainless steel autoclave and kept at 160 °C for 12 h. After naturally cooling to room temperature, the precipitates were collected by centrifugation, washed with deionized water and ethanol for 5 times each, and then vacuum dried at 80 °C for 12 h. The obtained precursors were finally annealed in air at 400, 500, and 600 °C for 8 h to produce electrode materials (assigned as C@Mn_2_O_3_-400, C@Mn_2_O_3_-500, and C@Mn_2_O_3_-600), respectively. The average yield of the products is 107.5 ± 2.5%, based on the conversation of Mn(Ac)_2_·4H_2_O to Mn_2_O_3_. The yield of higher than 100% can be attributed to the amorphous carbon coating onto the surfaces of the materials, which will be discussed later.

### 4.2. Dynamic Structure Evolution

The effect of reaction time on the structures of the microspheres was investigated by conducting the reactions for 1, 2, 6, 12, 18, and 24 h, respectively, and keeping the other parameters identical.

### 4.3. The Reaction Temperature Effect

The influence of reaction temperature tothe structures of the microspheres was also performed by reacting at 120, 140, 180, and 200 °C, respectively, and keeping other parameters identical.

### 4.4. Materials Characterization 

The composition and crystalline structure of the samples were characterized with X-ray diffraction (XRD, Smart Lab, Rigaku, Japan) using Cu Kα radiation at 40 kV and 200 mA. All of the XRD patterns were collected at room temperature with a 0.01° step in 2θ from 10 to 80°. The microstructures of the materials were analyzed by using field emission scanning electron microscopy (FESEM, JSM-7001F, 10 kV, Japan Electron Optics Laboratory, Tokyo, Japan) and transmission electron microscopy (TEM, JEOL, JEM-2100, 200 kV). In the preparation of samples for TEM observation, the materials were first dispersed in ethanol by using an ultrasonic bath for 10 min and then dropped onto copper grids, which were then dried in air at room temperature and kept in vacuum before TEM observation. Thermal analysis (TG-DTA) was performed with (TA-Q100) thermogravimetric analyzer under N_2_ atmosphere at a heating rate of 10 °C min^−1^ from room temperature to 1000 °C. The N_2_ adsorption and desorption isotherms were obtained by using a Belsorp-Mini adsorption apparatus (MicrotracBEL Corporation, Tokyo, Japan). The pore size distribution was determined by using BarreteJoynereHalenda (BJH) method that was applied to the desorption branch of adsorption-desorption isotherms. X-ray photoelectron spectroscopy (XPS) was collected on a Thermo Scientific Escalab 250Xi (ESCALAB 250Xi, Thermo Fisher Scientific, Waltham, MA, USA) with an Al Kα X-ray source and at 0.85 eV resolution. The chemical peaks that were obtained in the XPS spectra were corrected with respect to the C-1s peak at 284.6 eV. FTIR spectra were recorded with a Nicolet 5700 spectrometer (Thermo Nicolet Corporation, Verona, WI, USA) from KBr pellets with samples in the range of 500–4000 cm^−1^.

### 4.5. Electrochemical Properties of C@Mn_2_O_3_Microspheres

For the electrochemical measurements in a three electrode system, as-prepared active materials (C@Mn_2_O_3_-400, C@Mn_2_O_3_-500 or C@Mn_2_O_3_-600, 80 wt %) and AC (10 wt %) were first mixed together, and then grinded for ca. 10 min to obtain a mixture. Then, binder (polytetrafluoroethylene, PTFE, 10 wt %) was added into the mixture and mixed for 10 min. The mixture obtained was coated onto the surfaces of nickel meshes (1 × 1 cm^2^) subsequently. After dried at 100 °C for 12 h, the meshes with active materials (1.5 mg cm^−2^) were finally pressed under 10 MPa for 30 s to obtain working electrodes. The electrolyte used in the system was KOH aqueous solution (6 M). Platinum foil and saturated calomel electrode (SCE) were used as counter electrode and reference electrode, respectively. Cyclic Voltammogram (CV) was measured with an electrochemical workstation (CHI 660D, CH Instruments Inc., Shanghai, China). Galvanostatic charge/discharge cycle tests were performed on a LAND Cell test system (CT2001A, Wuhan, China). The specific capacitances of the supercapacitors can be evaluated from the charge/discharge tests with Equation (1):

*C_m_* = *I*∆*t*/*m*∆*V* (1)

where *C_m_* is the specific capacitance of the supercapacitor (F g^−1^), *I* is the current of the charge/discharge (A), and ∆*t* is the discharging time period in seconds for the potential change ∆*V*. The *m* is the loaded mass of active materials. All of the electrochemical measurements were carried out at room temperature.

### 4.6. Asymmetric Supercapacitors

The electrochemical performances of as-prepared materials were further evaluated in two-electrode cells consisting of C@Mn_2_O_3_-500 and AC to assemble asymmetric supercapacitors. The AC with a surface area of 1941 m^2^ g^−1^, a total pore volume of 0.96 cm^3^ g^−1^, and an average pore diameter of ca. 4 nm was applied as negative electrode materials to assemble asymmetric supercapacitors. The specific capacitance of AC electrodes is 221 F g^−1^ at current density of 0.5 A g^−1^, based on its galvanostatic charge/discharge curves with a potential window of −1–0 V in KOH electrolyte solution (6 M). The charge balance between the two electrodes follows the relationship *q*_+_ *= q*_−_. The charges stored by each of the electrodes depend on specific capacitance (*C*), potential range for the charge/discharge process (*V*), and the loaded total mass of active materials (*m*), following Equation (2).

*Q* = *C* × ∆*V* × *m* (2)

*m*_+_/*m*_−_ = (*C*_−_ × ∆*V*_−_)/(*C*_+_ × ∆*V*_+_) (3)

In order to get *q*_+_ *= q*_–_, the mass balancing will follow Equation (3). On the basis of specific capacitance values (*C*) and potential windows (*V*) that were found for the C@Mn_2_O_3_-500 microspheres and AC, respectively, the optimal mass ratio between the two electrodes is *m_C@Mn_2_O_3_-500/_m_AC_* = 0.45 in KOH (6 M) electrolytes for the asymmetric cells.

The energy (*E*) and power (*P*) densities of the asymmetric supercapacitors can be calculated with the following equations (Equations (4) and (5)).

*E* = 0.5*C*∆*V*^2^/3.6 (4)

*P* = *E* × 3600/∆*t* (5)

where *C* is the total capacitance (F g^−1^) of the cell that can be achieved in according to Equation (1), ∆*V* is the cell voltage (V), and ∆*t* is the discharge time (s).

## Supplementary Materials

The following are available online at www.mdpi.com/2079-4991/7/12/409/s1, Figure S1: (a–f) Low and (a1–f1) high magnification FESEM images and (a2–f2) size distributions of MnCO_3_ microspheres synthesized at 160 °C for 1, 2, 6, 12, 18 and 24 h, respectively, Figure S2: (a–f) Low, (a1–f1) high magnification FESEM images and (a2–f2) size distribution of MnCO_3_ microspheres synthesized at 120, 140, 160, 180 and 200 °C, respectively., Figure S3: XPS spectra of MnCO_3_ precursor and GC@Mn_2_O_3_ microspheres Surveys of (a) MnCO_3_ and (d) GC@Mn_2_O_3_ microspheres, Mn-2p core-level XPS spectra of (b) MnCO_3_ and (e) GC@Mn_2_O_3_, and O-1s core-level XPS spectra of (c) MnCO_3_ and (f) GC@Mn_2_O_3_., Figure S4: Electrochemical properties of electrodes made with active carbon. (a) CV curves at different scan rates, (b) ganvostatic charge/discharge curves at different current densities, (c) cycle stability at current density of 4 A g^-1^, and specific capacitances at different current densities, Figure S5: Electrochemical properties of electrodes made with monodispersed microspheres after annealed at 400 °C. (a) CV curves at different scan rates, (b) ganvostatic charge/discharge curves at different current densities, (c,d) cycle stability at current density of 4 A g^-1^, (e) specific capacitances at different current densities and (f) EIS spectrum., Figure S6: The electrochemical properties of electrodes made with monodispersed microspheres after annealed at 600 °C. (a) CV curves at different scan rates, (b) ganvostatic charge/discharge curves at different current densities, (c,d) cycle stability at current density of 2 A g^-1^, (e) specific capacitances at different current densities and (f) EIS spectrum.
