# Supplementary material for: Hierarchical Mn2O3 Microspheres In-Situ Coated with Carbon for Supercapacitors with Highly Enhanced Performances
Source: Nanomaterials (Basel). 2017 Nov 23;7(12):409. doi: 10.3390/nano7120409 (PMC5746899; doi:10.3390/nano7120409)
Supplement: Supplementary file 1 [file nanomaterials-07-00409-s001.pdf]

Hierarchical Mn_2O_3 Microspheres In-Situ Coated with Carbon for Supercapacitors with Highly Enhanced Performances

Feilong Gong [1,2], Shuang Lu [1], Lifang Peng [1], Jing Zhou [1], Jinming Kong [3], Dianzeng Jia [1] and Feng Li [1,2,4,*]

1. The Dynamic Structure Evolution of Precursor Microspheres

In order to investigate the formation of $MnCO_3$ precursor microspheres in the reactions, dynamic evolution (Figure S1) of the microsphere structures was performed by conducting the reaction from 1 to 24 hours. It was found that sphere-like particles of 1.35 ± 0.36 μm in diameter together with a lot of small nanoparticles (Figure S1a) were generated after reacting for 1 hour. $MnCO_3$ nanoparticles, which nucleate rapidly in the reaction, act as seeds to grow sphere-like particles with rough surfaces. After reacting for 2 hours, however, microspheres of 1.95 ± 0.47 μm (Figure S1b) were produced in the reaction on a large scale, and the smaller nanoparticles completely disappeared in the system. The microspheres further grown into bigger in diameter of 2.05 ± 0.37 μm (Figure S1c) and 3.96 ± 0.55 μm (Figure S1d) after prolonging the reaction time to 6 and 12 hours, respectively. The bigger microspheres also possess smoother surfaces in consuming of smaller nanoparticles. The Ostwald ripening mechanism can be applied to explain the disappearances of smaller nanoparticles with higher energy and the growth of microspheres with bigger size and smoother surfaces. The uniformity of the microspheres can be improved dramatically after performing the reaction for 12 hours. However, the microspheres with smaller diameters and rougher surfaces were again produced after further conducting the reactions for 18 and 24 hours as shown in Figure S1e-f, respectively.

The precursor microsphereswith different diameters as shown in Figure S2 can be also produced on a large scale by performing reaction at 120, 140, 180 and 200 °C, respectively. It is convenient for us to control the sizes of $MnCO_3$ microspheres by simply adjusting the reaction time and temperature.

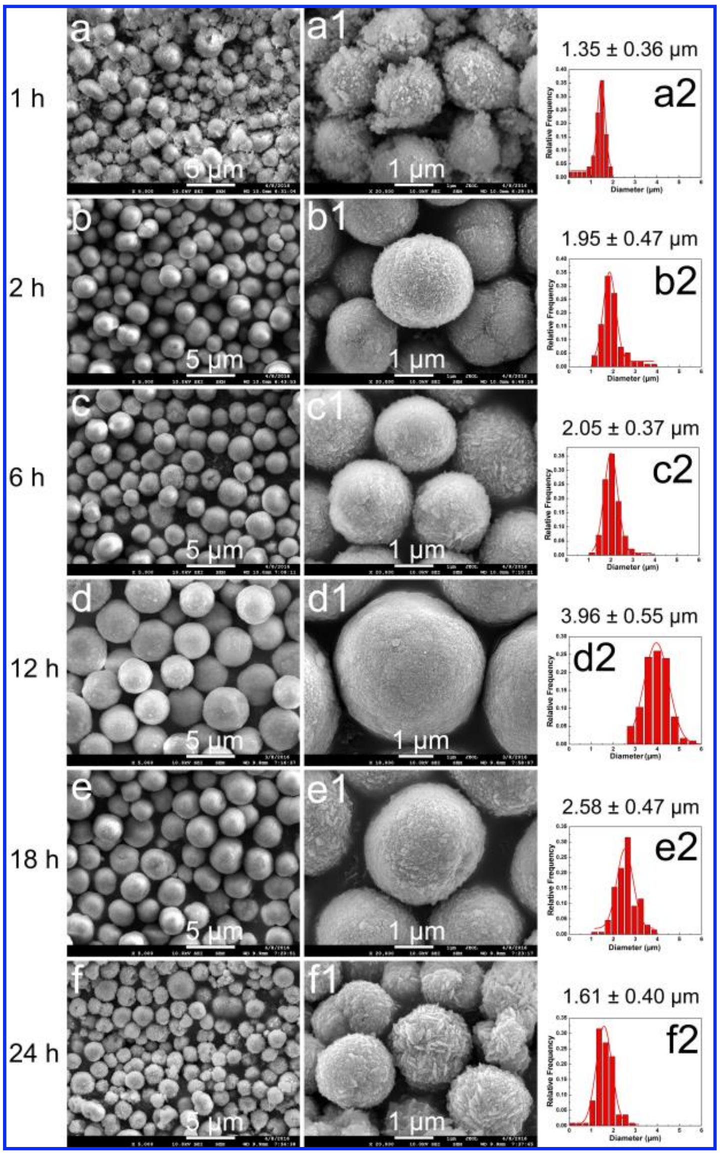

Figure S1. (**a**–**f**) Low and (**a1**–**f1**) high magnification FESEM images and (**a2**–**f2**) size distributions of $MnCO_3$ microspheres synthesized at 160 °C for 1, 2, 6, 12, 18 and 24 h, respectively.

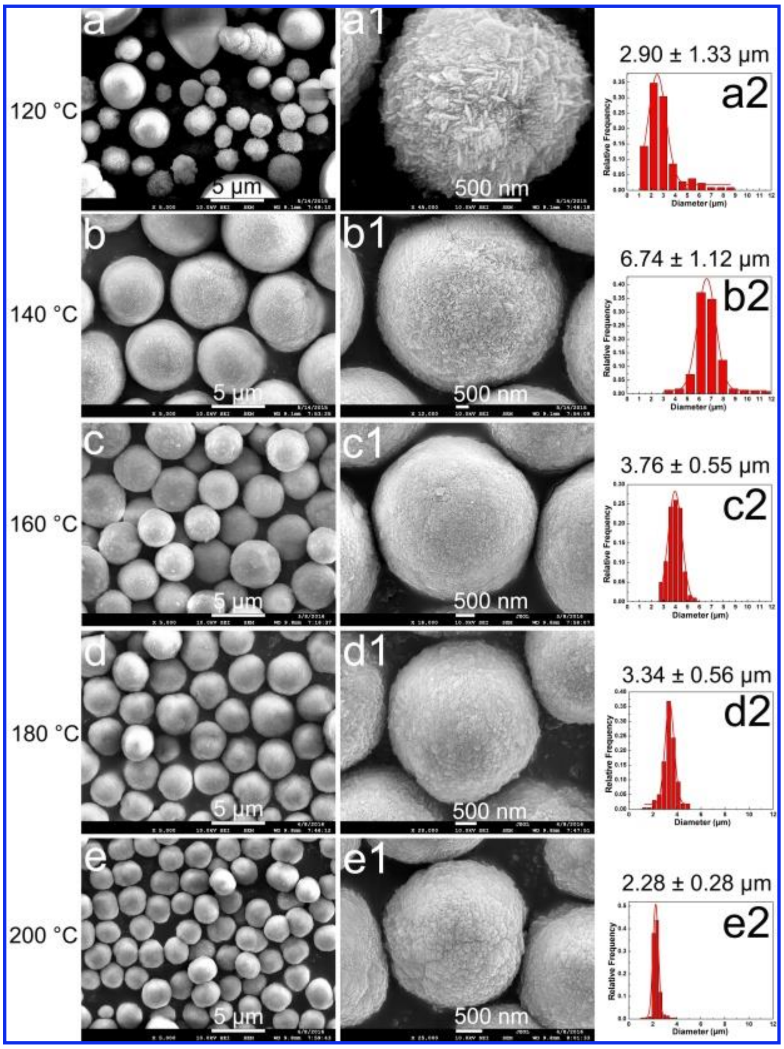

Figure S2. (**a**–**f**) Low, (a1–f1) high magnification FESEM images and (**a2**–**f2**) size distribution of $MnCO_3$ microspheres synthesized at 120, 140, 160, 180 and 200 °C, respectively.

2. Xps Spectra of Precursor Microspheres before and after Annealed At 500 °C

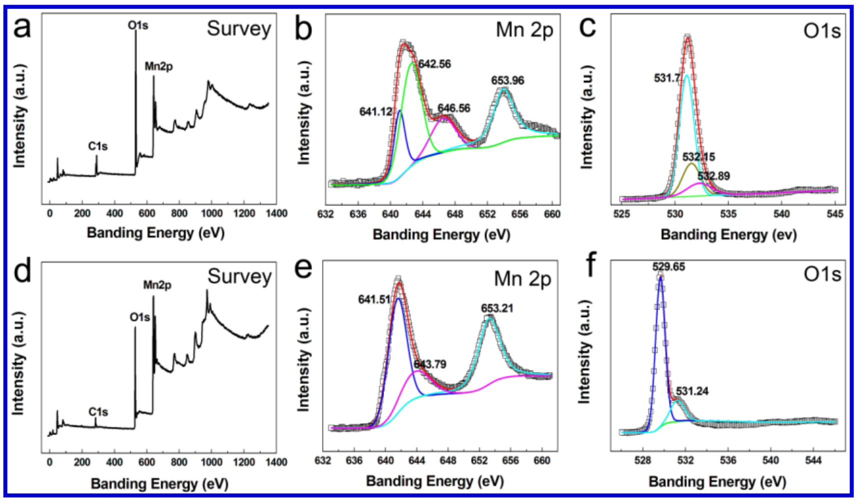

Figure S3.XPS spectra of $MnCO_3$ precursor and GC@Mn_2O_3 microspheres Surveys of (**a**) $MnCO_3$ and (**d**) GC@Mn_2O_3 microspheres, Mn-2p core-level XPS spectra of (**b**) $MnCO_3$ and (**e**) GC@Mn_2O_3, and O-1s core-level XPS spectra of (**c**) $MnCO_3$ and (**f**) GC@Mn_2O_3.

3. Electrochemical Properties of Active Carbon (Ac)

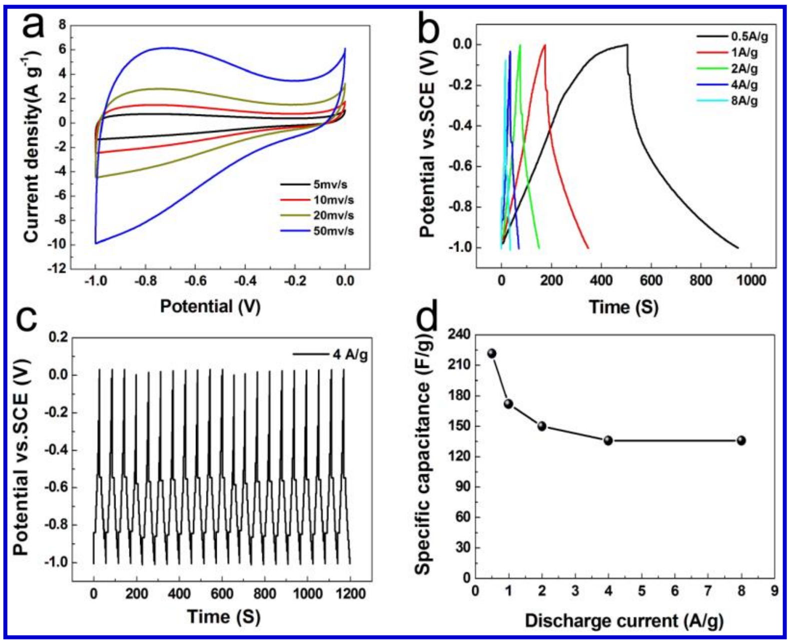

Figure S4. Electrochemical properties of electrodes made with active carbon. (**a**) CV curves at different scan rates, (**b**) ganvostatic charge/discharge curves at different current densities, (**c**) cycle stability at current density of 4 A g^{-1}, and specific capacitances at different current densities.

4. Electrochemical Properties of Materials After Annealed at 400 °C

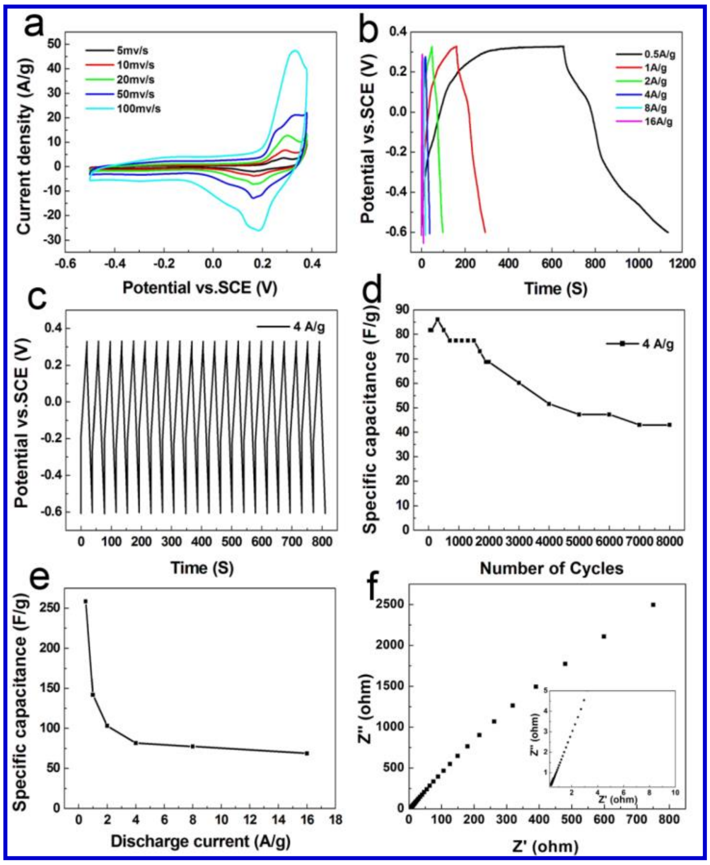

Figure S5. Electrochemical properties of electrodes made with monodispersed microspheres after annealed at 400 °C. (**a**) CV curves at different scan rates, (**b**) ganvostatic charge/discharge curves at

different current densities, (**c,d**) cycle stability at current density of 4 A g-1,(**e**) specific capacitances at different current densities and (**f**) EIS spectrum.

5. Electrochemical Properties of Materials After Annealed At 600 °C

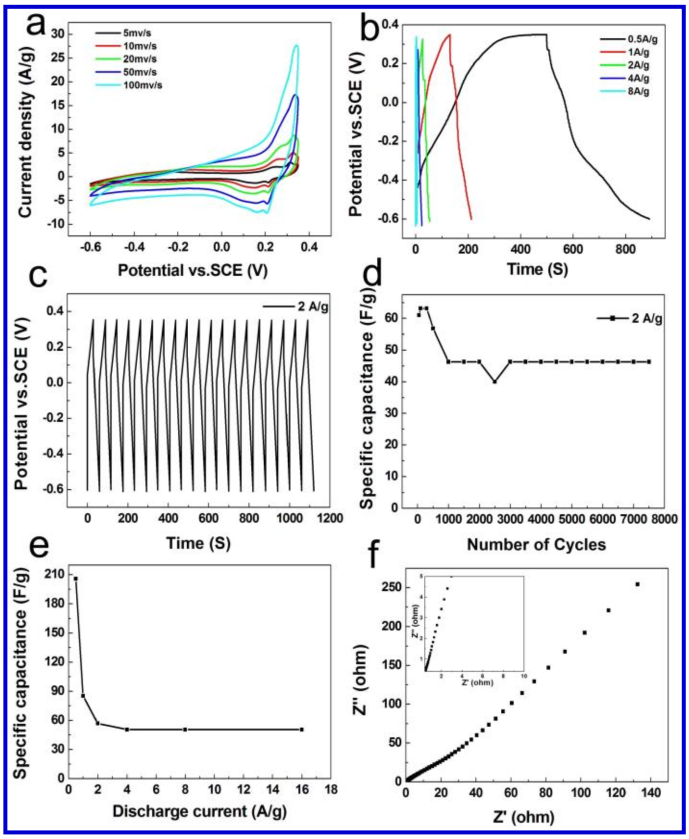

Figure S6. The electrochemical properties of electrodes made with monodispersed microspheres after annealed at 600 °C. (**a**) CV curves at different scan rates, (**b**) ganvostatic charge/discharge curves at different current densities, (**c,d**) cycle stability at current density of 2 A g-1, (**e**) specific capacitances at different current densities and (**f**) EIS spectrum.
